# Retrospective study on constructing a nomogram model based on clinical data to predict the recurrence probability of patients with intrauterine adhesions after separation surgery

**DOI:** 10.3389/fmed.2026.1654189

**Published:** 2026-01-30

**Authors:** Qin Wan, Ming Zhou, Chen Qian, Xiaohong Zhang

**Affiliations:** Department of Gynaecology and Obstetrics, Xuancheng Central Hospital, Xuancheng, Anhui, China

**Keywords:** decision curve, hysteroscopic adhesiolysis, intrauterine adhesions, nomogram model, postoperative recurrence, risk factors

## Abstract

**Background:**

Intrauterine adhesions (IUAs) are a common gynecological condition that can lead to menstrual abnormalities, infertility, and recurrent pregnancy loss. Hysteroscopic adhesiolysis is the standard treatment, but recurrence rates remain high, and factors influencing recurrence are not fully understood. This study aims to develop and validate a nomogram based on clinical data for predicting the probability of recurrence in patients with IUAs following hysteroscopic adhesiolysis.

**Methods:**

A retrospective analysis included 256 patients who underwent hysteroscopic adhesiolysis at our hospital between October 2018 and October 2020, forming the modeling group. Additionally, 128 patients undergoing the same procedure between November 2020 and September 2021 served as the validation group. Patients in both groups were divided into recurrence and non-recurrence subgroups based on whether intrauterine adhesions recurred post-surgery. Within the modeling group, multivariate logistic regression identified factors associated with recurrence, which were incorporated into a nomogram. Model performance was evaluated using ROC and calibration curves, with external validation conducted in the validation group.

**Results:**

In the modeling group, the recurrence rate after the separation of intrauterine adhesions was 20.31%; Multivariate logistic regression analysis showed that disease duration >12 months and 12 days, number of induced abortions >2, number of prior uterine cavity operations >1, and adhesion range ≥1/2 of the cavity were independent risk factors for the recurrence (*p* < 0.05); Internal validation showed good calibration according to the Hosmer–Lemeshow (H–L) test, *χ*^2^ = 6.427, *p* = 0.316, and the area under the ROC curve was 0.767. External validation showed similar good calibration, *χ*^2^ = 7.006, *p* = 0.352; the AUC was 0.779. The nomogram demonstrated high clinical utility for predicting postoperative recurrence of intrauterine adhesions across threshold probabilities of 0.02–0.89 in the modeling group and 0.02–0.92 in the validation group.

**Conclusion:**

Disease duration >12 months and 12 days, more than two induced abortions, more than one preoperative intrauterine procedure, and an adhesion range ≥1/2 were identified as independent risk factors for postoperative recurrence in patients with intrauterine adhesions. The nomogram prediction model developed based on these factors provides clinicians with a simple and intuitive tool to individualize the prediction of recurrence risk after adhesiolysis.

## 1 Introduction

Intrauterine adhesions (IUAs) are a common gynecological condition in clinical practice, primarily caused by damage to the endometrial basal layer (1, 2). In recent years, clinical decision support systems (CDSS) have shown significant potential in risk prediction and individualized treatment planning. Prediction models such as nomograms can serve as part of CDSS, assisting clinicians in evaluating the risk of recurrence of IUA and formulating personalized intervention strategies (3). IUAs mainly manifest as preterm birth, secondary infertility, reduced menstruation, and other reproductive function and menstrual abnormalities (4, 5). Intrauterine operations (particularly those involving curettage, such as induced abortions and uterine surgery) are the main causes of uterine adhesions and endometrial damage leading to IUAs, and the incidence of IUAs has shown a rising trend in recent years, reaching as high as 43% among infertile women, and poses a significant threat to both reproductive health and overall physical and psychological well-being (6, 7). Currently, hysteroscopic adhesiolysis is the main method for clinically treating uterine adhesions, capable of accurately and intuitively separating the adhesion bands, reducing the loss of normal endometrium, and promoting the recovery of the uterine cavity shape (8). However, the recurrence rate after hysteroscopic adhesiolysis is relatively high, and the factors affecting recurrence are not yet fully understood. Therefore, this study collected patient data, analyzed the factors influencing recurrence after hysteroscopic adhesiolysis, and constructed a nomogram to provide references for clinical treatment and postoperative care, aiming to reduce the recurrence rate.

## 2 Materials and methods

### 2.1 Study design and participants

A retrospective analysis was conducted on patients who underwent hysteroscopic adhesiolysis at our hospital from October 2018 to September 2021. This study was approved by the Ethics Committee of Xuancheng Central Hospital. Inclusion criteria: (1) Patients diagnosed with uterine adhesions through hysteroscopy before surgery; (2) Patients with a history of sexual activity; (3) Patients without contraindications for hysteroscopic surgery; (4) The severity of adhesions was classified as Grade I–II in all cases (9). (5) Patients who signed an informed consent form. Exclusion criteria: (1) Patients with coagulation disorders; (2) Patients unable to undergo hysteroscopy due to excessive uterine curvature; (3) Patients with cervical adhesions; (4) Patients with consciousness disorders or mental illnesses; (5) Patients with tuberculous uterine adhesions.

### 2.2 Surgical method

All patients underwent hysteroscopic adhesiolysis. Preoperatively, vaginal cleansing was performed 3 days before surgery, and patients were instructed to fast with water restriction starting 6 h prior to surgery. Misoprostol 600 μg was inserted into the vagina 2 h before surgery for cervical softening. After anesthesia, the patient was positioned in the lithotomy position. The surgical area was disinfected, and the cervical canal was dilated to a size of 10–12. Physiological saline was injected into the uterus at a rate of 260–450 mL/min to expand it, while maintaining a pressure of 100–150 mmHg. Depending on the location, severity, and density of adhesions, separation was performed using hysteroscopic electrodes, micro-scissors, or direct-tip separation to avoid damaging the endometrium. Postoperative oral antibiotics were prescribed for 1 week to prevent infection. All procedures in this study were conducted by a single experienced surgeon at the same medical center.

### 2.3 Data collection

This study obtained the required data through a retrospective review of the hospital’s electronic medical record system and medical documentation system. Data collection was conducted by three clinical nurses from the research team, all of whom possessed full-time bachelor’s degrees and had at least 5 years of experience in obstetrics and gynecology. To ensure consistency, the three data collectors independently extracted records for the first five subjects in both the recurrence and non-recurrence groups. Any discrepancies were then discussed, and the first author and corresponding author standardized the data collection criteria. Data collection was finalized once consensus was reached within the research team. Collected data included the number of preoperative intrauterine procedures (i.e., intrauterine surgery performed due to underlying disease), the number of pregnancies and deliveries, preoperative menstrual conditions, the number of pregnancy terminations, the duration of illness (based on a comprehensive assessment of medical history, symptoms, and auxiliary examinations), and age. The extent and nature of uterine adhesions were assessed by hysteroscopy, along with postoperative placement of an intrauterine device (not placed if postoperative recovery was good and no intrauterine infection occurred; otherwise, it was placed) and the duration of surgery.

### 2.4 Follow-up

Follow-up was conducted for 1 year post-surgery through phone calls, both outpatient and inpatient visits. The follow-up period for the modeling group ended in October 2021, and for the validation group in September 2022. The follow-up rate was 100%. Monthly follow-ups were conducted for three consecutive months, followed by quarterly follow-ups. Patients’ menstrual recovery and recurrence status (after postoperative menstrual improvement, hypomenorrhea or amenorrhea reappeared, with definite adhesion formation and AFS score ≥1) were assessed. Patients who experienced recurrence were instructed to seek timely medical consultation.

### 2.5 Statistical analysis

SPSS 25.0 software was used for the statistical analysis of the data in this study. Quantitative data were expressed as ( $\bar{x}\pm s$ , mean ± standard deviation) and analyzed using the *t*-test; categorical data were expressed as cases (%) and analyzed using the Chi-square test (*χ*^2^). The Receiver Operating Characteristic (ROC) curve analysis was employed to assess the optimal cutoff value for disease duration. In the modeling group, multivariate logistic regression analysis was used to identify factors affecting postoperative recurrence. The prediction model, constructed using nomograms, was developed using R software. The model’s predictive validity was evaluated using the Hosmer–Lemeshow (H–L) goodness-of-fit test, and its discriminative ability was assessed using the ROC curve. The clinical utility of the model was evaluated using decision curve analysis (DCA). A *p*-value of <0.05 was considered statistically significant.

## 3 Results

### 3.1 Basic information of patients and incidence of IUAs

**Figure 1:**
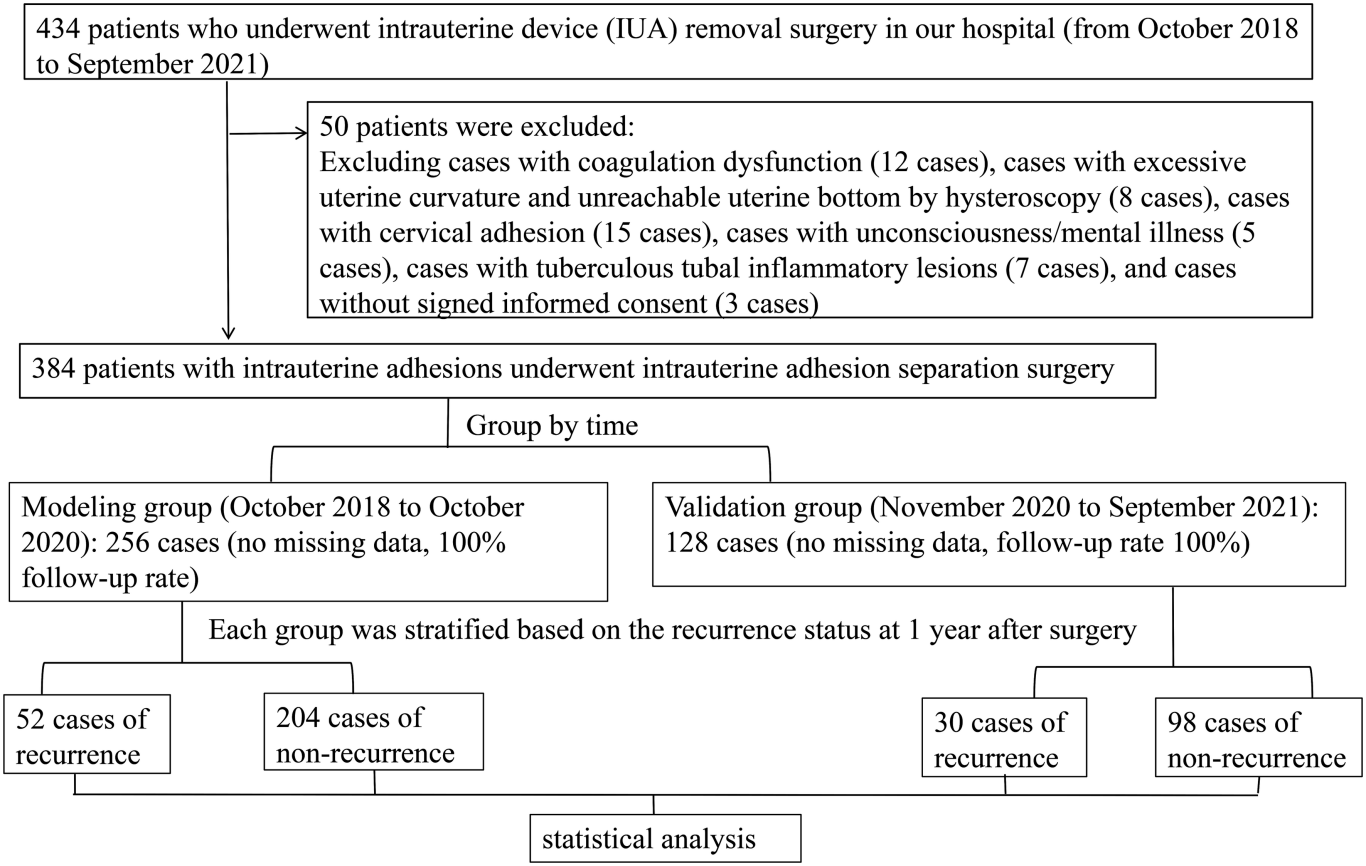
Flowchart of case collection.

A retrospective review was conducted of 434 patients who underwent hysteroscopic adhesiolysis for intrauterine adhesions at our hospital between October 2018 and September 2021. Among these patients, 12 with coagulation disorders, 8 in whom the hysteroscope could not reach the uterine fundus due to excessive uterine curvature, 15 with cervical adhesions, 5 with impaired consciousness or psychiatric disorders, 7 with tuberculous intrauterine adhesions, and 3 who did not provide written informed consent were excluded. Ultimately, 384 patients were included in the present study. Patients were grouped according to the time of surgery: the modeling group comprised patients who underwent surgery between October 2018 and October 2020 (*n* = 256), and the validation group comprised patients treated between November 2020 and September 2021 (*n* = 128). There were no missing data in either group, and the follow-up rate was 100%. The postoperative recurrence rate of intrauterine adhesions after hysteroscopic adhesiolysis was 20.31% (52/256) in the modeling group and 23.44% (30/128) in the validation group. See Figure 1.

### 3.2 Comparison of baseline characteristics between the modeling and validation groups

To ensure the validity of external validation, baseline characteristics of patients in the modeling and validation groups were compared (Table 1). The results showed no statistically significant differences between the two groups in disease duration, number of induced abortions, number of preoperative intrauterine procedures, extent of adhesions, or postoperative intrauterine device placement (all *p* > 0.05), indicating good homogeneity between the groups and suitability for external validation.

**Table 1:** Comparison of baseline data between modeling group and validation group.

Project	Modeling group (*n* = 256)	Validation group (*n* = 128)	*t*/*χ*^2^	*P*
Duration of illness (months)	13.66 ± 2.87	13.91 ± 2.66	0.824	0.410
Number of abortions	1.82 ± 0.55	1.76 ± 0.57	0.996	0.320
Number of preoperative intrauterine cavity operations (times)	1.20 ± 0.35	1.27 ± 0.40	1.760	0.079
Extent of adhesion				
<1/2	188 (73.44)	94 (26.56)	0.000	1.000
≥1/2	68 (73.44)	34 (26.56)		
Insertion of an IUD after surgery				
Yes	198 (77.34)	89 (22.66)	2.759	0.097
No	58 (69.53)	39 (30.47)		

### 3.3 Comparison of clinical data between recurrence and non-recurrence groups in the modeling group

The postoperative recurrence rate of uterine adhesion separation in the modeling group was 20.31% (52/256). There were statistically significant differences between the two groups in terms of duration of illness, number of induced abortions, number of preoperative intrauterine procedures, extent of adhesions, and postoperative intrauterine device placement (*p* < 0.05). However, there were no significant differences in the number of pregnancies, number of deliveries, age, preoperative menstrual conditions, nature of adhesions, and operation time between the two groups (*p* > 0.05). See Table 2.

**Table 2:** Comparison of clinical data between the recurrence group and the non-recurrence group in the modeling group (*n*, %).

Project	*N*	Recurrence group (*n* = 52)	Non-recurrence group (*n* = 204)	*t*/*χ*^2^	*P*
Pregnancy (times)		4.17 ± 1.25	3.98 ± 1.20	1.011	0.313
Parity (times)		2.66 ± 0.75	2.49 ± 0.70	1.541	0.125
Age (years)		33.21 ± 8.36	31.58 ± 8.02	1.297	0.196
Duration of illness (months)		15.19 ± 4.05	13.27 ± 3.64	3.317	0.001
Number of abortions		2.35 ± 0.72	1.69 ± 0.53	7.412	0.000
Preoperative menstrual status					
Normal	168	36 (21.43)	132 (78.57)	0.376	0.540
Abnormal	88	16 (18.18)	72 (81.82)		
Number of preoperative intrauterine cavity operations (times)		1.52 ± 0.48	1.12 ± 0.35	6.782	0.000
Extent of adhesion					
<1/2	188	30 (15.96)	158 (84.04)	8.293	0.004
≥1/2	68	22 (32.35)	46 (67.65)		
Adhesion properties					
Compactness	153	32 (20.92)	121 (79.08)	0.328	0.849
Membrane	41	7 (17.07)	34 (82.93)		
Muscular	62	13 (25.00)	49 (79.3)		
Insertion of an IUD after surgery					
Yes	198	34 (17.17)	164 (82.83)	5.326	0.021
No	58	18 (31.03)	40 (68.97)		
Operative time (min)		22.09 ± 5.71	20.85 ± 5.32	1.478	0.141

### 3.4 Multivariate logistic regression analysis of factors influencing recurrence after hysteroscopic adhesiolysis

Variables with *p* < 0.05 in Table 2—including disease duration, number of induced abortions, number of preoperative intrauterine procedures, extent of adhesion, and postoperative intrauterine device (IUD) placement—were included in the multivariate logistic regression analysis using a stepwise forward method. The results showed that a disease duration >12 months and 12 days, more than two induced abortions, more than one preoperative intrauterine procedure, and an adhesion extent ≥1/2 were independent risk factors for recurrence after hysteroscopic adhesiolysis (*p* < 0.05). See Table 3.

**Table 3:** Multivariate logistic regression analysis of factors influencing recurrence of intrauterine adhesion dissection.

Project	*B*	Wald	SE	*P*	OR	95%CI
The duration of the disease >12 months and 12 days	0.900	0.401	5.028	0.025	2.459	1.120–5.399
The number of abortions >2	1.717	0.369	21.598	0.000	5.565	2.698–11.478
Preoperative intrauterine cavity manipulation >1 time	0.934	0.365	6.555	0.010	2.545	1.245–5.204
Adhesion range ≥1/2	1.076	0.371	8.420	0.004	2.934	1.418–6.071
constant	−3.206	0.427	56.441	0.000	0.014	–

The optimal cutoff value for disease duration determined by ROC curve analysis was 12 months and 12 days, with a sensitivity of 78.58% and a specificity of 86.77%.

### 3.5 Construction of a nomogram for postoperative recurrence of uterine adhesions

**Figure 2:**
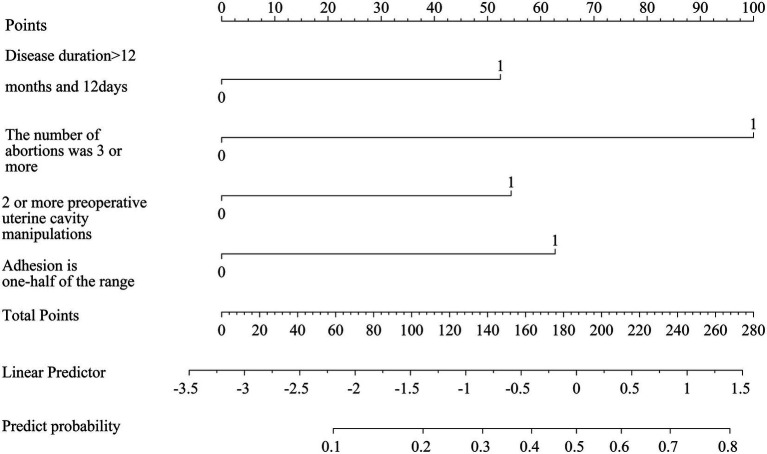
Construction of a nomogram of recurrence after intrauterine adhesion dissection.

A nomogram for postoperative recurrence of uterine adhesions was constructed using the results of the multivariate logistic regression analysis with R software. A duration of illness >12 months and 12 days scored 52.5 points, more than 2 induced abortions scored 100 points, more than 1 preoperative intrauterine operation scored 53 points, and an adhesion range ≥1/2 scored 62.5 points. See Figure 2.

### 3.6 Internal validation of the predictive model for postoperative recurrence of uterine adhesions

**Figure 3:**
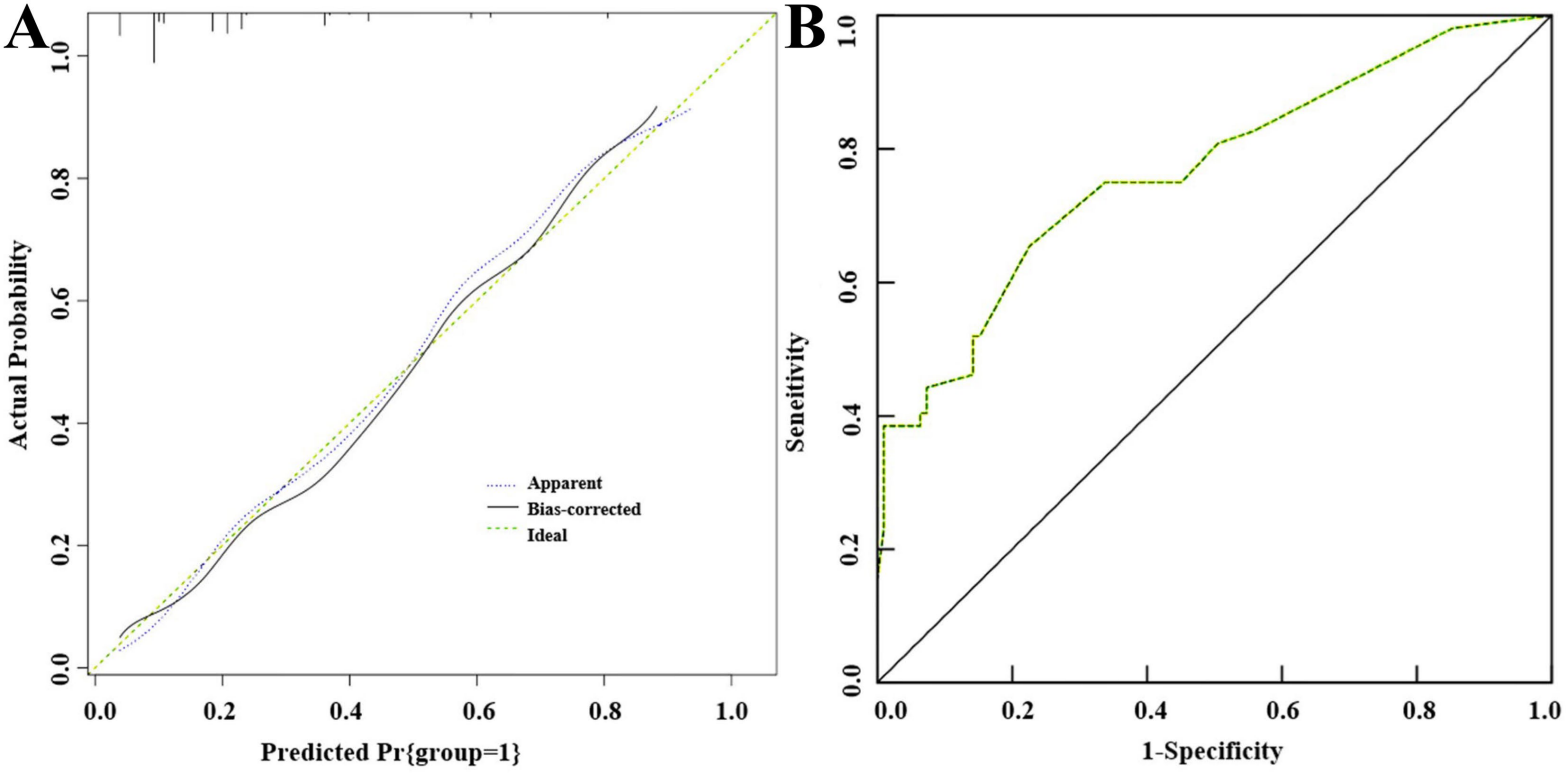
Internal validation of nomogram prediction model for recurrence after intrauterine adhesion separation: **(A)** H–L fit curve; **(B)** ROC curve.

The predictive validity of the model was assessed using the Hosmer–Lemeshow (H–L) goodness-of-fit test. The results showed good consistency between predicted and actual values (Figure 3A), with *χ*^2^ = 6.427, *p* = 0.316. The discriminative ability of the predictive model was evaluated using the ROC curve, showing an area under the curve of 0.767 (95%CI: 0.689–0.845), with specificity and sensitivity of 77.45 and 65.38%, respectively (Figure 3B).

### 3.7 External validation of the predictive model for postoperative recurrence of uterine adhesions

**Figure 4:**
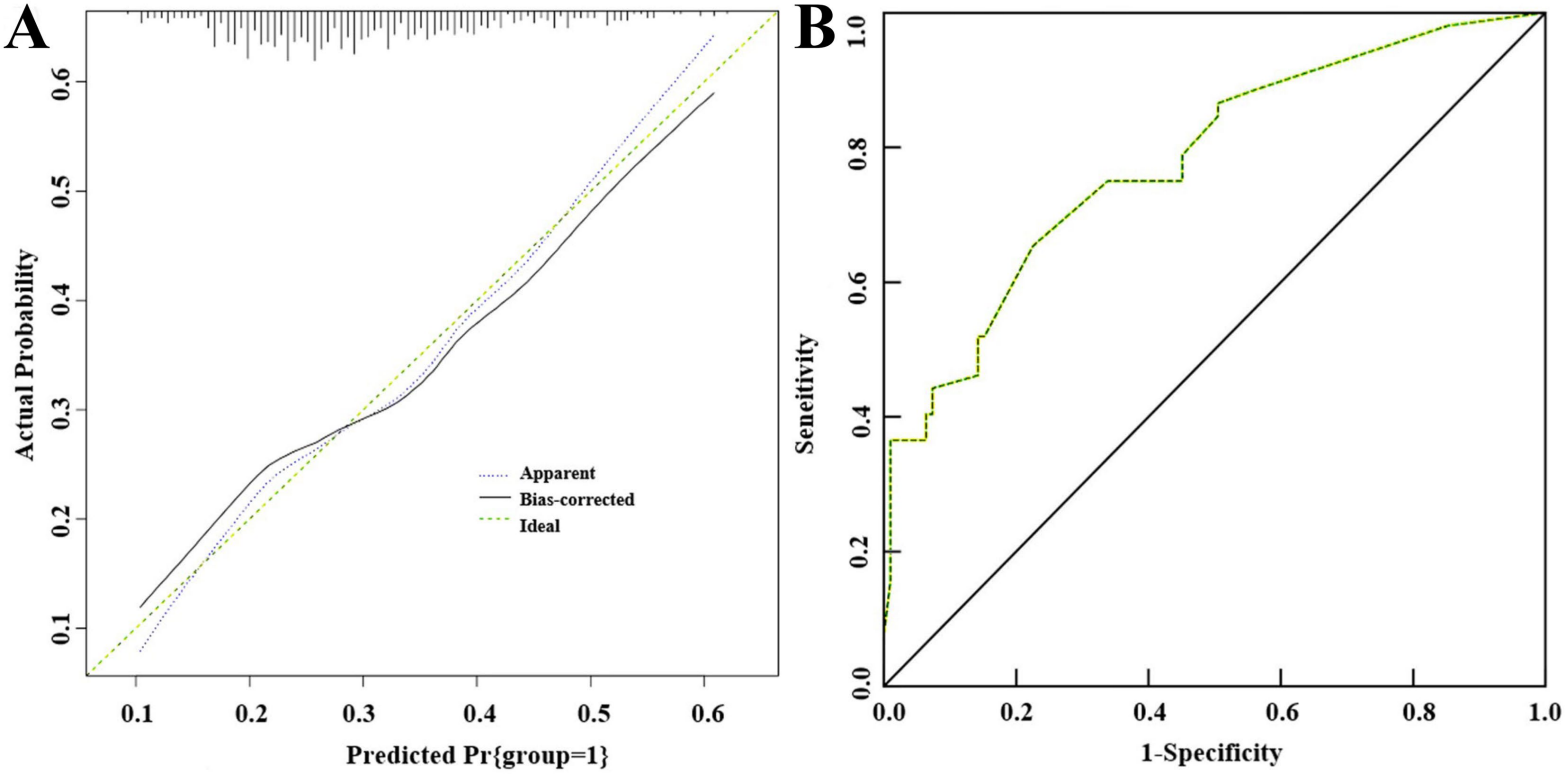
External validation of nomogram prediction model for recurrence after intrauterine adhesion separation. **(A)** H–L fit curve; **(B)** ROC curve.

The postoperative recurrence rate of uterine adhesion separation in the validation group was 23.44% (30/128). There were statistically significant differences between the two groups in terms of duration of illness, number of induced abortions, number of preoperative intrauterine procedures, adhesion range, and whether insertion of an IUD after surgery (*p* < 0.05). There were no statistically significant differences between the two groups in terms of gravidity, parity, age, preoperative menstrual status, adhesion characteristics, and operation time (*p* > 0.05), as shown in Table 4. External validation results demonstrated good consistency between predicted and actual values in the H–L goodness-of-fit test, with *χ*^2^ = 7.006, *p* = 0.352 (Figure 4A); the area under the ROC curve was 0.779 (95%CI: 0.706–0.852), with specificity and sensitivity of 77.50 and 65.40%, respectively (Figure 4B).

**Table 4:** Comparison of clinical data between the recurrence group and the non-recurrence group in the validation group (*n*, %).

Project	*N*	Recurrence group (*n* = 30)	Non-recurrence group (*n* = 98)	*t*/*χ*^2^	*P*
Pregnancy (times)		4.19 ± 1.17	4.05 ± 1.02	0.635	0.526
Parity (times)		2.67 ± 0.71	2.54 ± 0.73	0.859	0.392
Age (years)		33.39 ± 7.75	31.17 ± 7.93	1.349	0.180
Duration of illness (months)		15.87 ± 4.10	13.31 ± 3.68	3.245	0.002
Number of abortions		2.29 ± 0.71	1.60 ± 0.50	5.954	0.000
Preoperative menstrual status				0.057	0.811
Normal	83	20 (24.10)	63 (75.90)		
Abnormal	45	10 (22.22)	35 (77.78)		
Number of preoperative intrauterine cavity operations (times)		1.56 ± 0.51	1.18 ± 0.37	4.480	0.000
Extent of adhesion					
<1/2	94	17 (18.09)	77 (81.91)	5.650	0.017
≥1/2	34	13 (38.24)	21 (61.76)		
Adhesion properties				1.519	0.468
Compactness	78	21 (26.92)	57 (73.08)		
Membrane	20	3 (15.00)	17 (85.00)		
Muscular	30	6 (20.00)	24 (80.00)		
Insertion of an IUD after surgery				9.669	0.002
Yes	89	14 (15.73)	75 (84.27)		
No	39	16 (41.03)	23 (58.97)		
Operative time (min)		21.88 ± 5.42	20.96 ± 5.33	0.824	0.411

### 3.8 Decision curve analysis (DCA) in the modeling and validation groups

**Figure 5:**
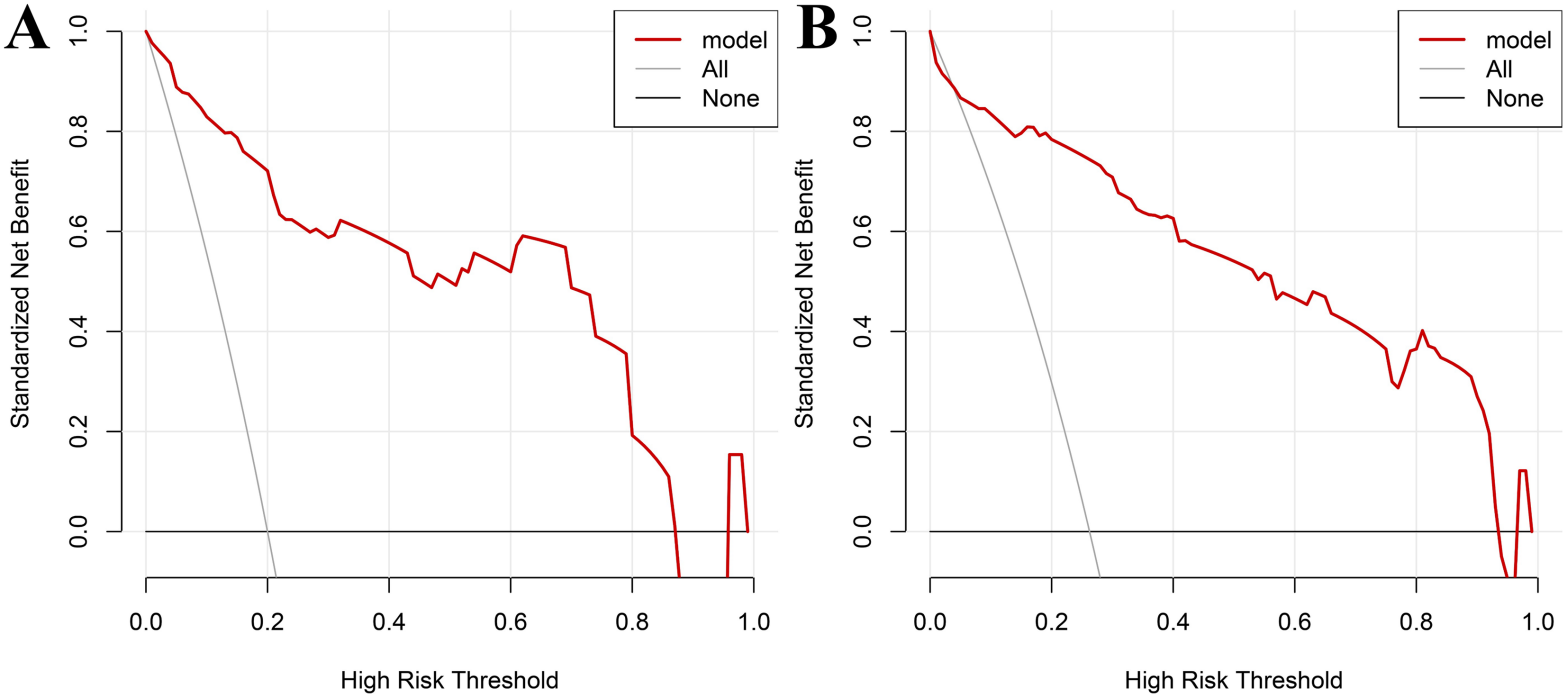
Decision curve analysis (DCA) in the modeling and validation groups: **(A)** DCA for the modeling group; **(B)** DCA for validation group.

Decision curve analysis was performed with threshold probability on the *x*-axis and net benefit on the *y*-axis. In the modeling group, the nomogram demonstrated high clinical utility for predicting postoperative recurrence of intrauterine adhesions when the threshold probability ranged from 0.02 to 0.89 (Figure 5A). In the validation group, the nomogram similarly showed high clinical utility across threshold probabilities of 0.02–0.92 (Figure 5B).

## 4 Discussion

Currently, hysteroscopic adhesiolysis is the primary clinical treatment for uterine adhesions. With ongoing technological advancements, this surgical technique has achieved high success rates. However, postoperative recurrence remains a significant clinical challenge (10, 11). Multiple studies have demonstrated that the recurrence rate of uterine adhesions is relatively high, varying between 20 and 50% (12, 13). A high recurrence rate not only offsets the short-term benefits of surgery but may also lead to long-term adverse outcomes such as abnormal menstrual patterns, infertility, and recurrent miscarriage (14). Therefore, identifying the factors that influence recurrence and implementing appropriate preventive measures is critical for improving patient prognosis.

In this study, patient data were collected for univariate analysis, which showed that the recurrence of uterine adhesions after separation surgery is related to the duration of illness, number of induced abortions, number of preoperative intrauterine procedures, adhesion range, and whether an intrauterine device was placed postoperatively. However, it was unrelated to the number of pregnancies, number of deliveries, age, preoperative menstrual conditions, the nature of adhesions, and operation time. Further multivariate logistic regression analysis indicated that a duration of illness >12 months and 12 days, more than two induced abortions, more than one preoperative intrauterine procedure, and an adhesion range ≥1/2 are independent risk factors for recurrence after uterine adhesion separation surgery. Analyzing the reasons, patients with longer disease duration tend to have poorer prognosis. Chronic long-term inflammation and abnormal fibrotic repair are the core pathological processes. Continuous damage to the endometrial basal layer can lead to persistent overexpression of profibrotic factors such as transforming growth factor-β1, which promotes myofibroblast differentiation and excessive extracellular matrix deposition, resulting in irreversible fibrotic scarring that severely impairs endometrial regeneration and receptivity (15–17). Multiple induced abortions and intrauterine procedures can directly damage the endometrial functional layer and even the basal layer through repeated physical trauma. Each procedure may exacerbate inflammatory responses and activate the aforementioned fibrotic pathways, while potentially depleting local endometrial stem cells with regenerative potential, thereby increasing the likelihood of intrauterine adhesion recurrence (8, 18). The extent of adhesions is closely related to the degree of endometrial damage; the broader the range, the more difficult it is for the endometrium to repair, significantly impacting the prognosis. However, the multivariate logistic regression analysis showed that postoperative IUD placement was not an influencing factor for recurrence after hysteroscopic adhesiolysis, which is inconsistent with the findings of previous studies (19). This discrepancy may be due to the relatively small sample size and the single-center nature of this study. In addition, a meta-analysis indicated that barrier agents such as balloon stents or hyaluronic acid gels may show a superior trend in preventing re-adhesion compared to IUDs (20). Therefore, the preventive effect of IUDs may not be absolute, and their efficacy could be influenced by patient populations, surgical techniques, and adjunctive treatment strategies. Larger prospective studies are needed in the future to clarify these findings.

The nomogram enables visualization and pictorial representation of multivariate analysis results, allowing for a more intuitive prediction of the recurrence risk after uterine adhesion separation surgery. Therefore, this study also constructed a nomogram based on the results of multivariate analysis to depict the factors influencing postoperative recurrence. For instance, if a patient’s disease duration exceeds 12 months and 12 days (scoring 52.5 points) and she has undergone more than two induced abortions (scoring 100 points), the total score would be 152.5 points, corresponding to a predicted recurrence probability of 0.35 as indicated at the bottom of the nomogram. Additionally, this study evaluated the discriminative ability and predictive validity of the model using ROC curves and Hosmer–Lemeshow (H–L) goodness-of-fit tests. Both internal and external validation results demonstrated good discriminative ability and predictive value of the model. Further decision curve analysis (DCA) demonstrated that the nomogram model had high clinical utility for predicting postoperative recurrence of intrauterine adhesions when the high-risk threshold probability ranged from 0.02 to 0.89 in the training cohort and from 0.02 to 0.92 in the validation cohort. Clinicians and healthcare providers can use various factors to predict the risk of recurrence after hysteroscopic adhesiolysis and implement early interventions accordingly.

In addition, the nomogram established in this study may serve as an essential component of a Clinical Decision Support System (CDSS) to assist clinicians in identifying high-risk patients and optimizing postoperative follow-up strategies. Integrating such predictive tools into CDSS platforms could enhance clinical decision-making and promote individualized care in gynecology.

In summary, a disease duration exceeding 12 months and 12 days, more than two induced abortions, more than one preoperative intrauterine procedure, and an adhesion extent of ≥1/2 are independent risk factors for recurrence after uterine adhesion separation surgery. The constructed nomogram provides a simpler and more intuitive means to predict postoperative recurrence. However, this study is a retrospective study with a relatively small sample size and without multicenter validation, which may limit the generalizability of the results. Future research will involve prospective studies with larger sample sizes and multicenter validation.

## Data Availability

The original contributions presented in the study are included in the article/supplementary material, further inquiries can be directed to the corresponding author/s.
